# Nicotinic acid promotes sleep through prostaglandin synthesis in mice

**DOI:** 10.1038/s41598-019-53648-7

**Published:** 2019-11-19

**Authors:** Éva Szentirmai, Levente Kapás

**Affiliations:** 1 Elson S. Floyd College of Medicine, Department of Biomedical Sciences, Washington State University, Spokane, Washington United States of America; 2 Sleep and Performance Research Center, Washington State University, Spokane, Washington United States of America

**Keywords:** Neurophysiology, Circadian rhythms and sleep

## Abstract

Nicotinic acid has been used for decades for its antiatherogenic properties in humans. Its actions on lipid metabolism intersect with multiple sleep regulatory mechanisms, but its effects on sleep have never been documented. For the first time, we investigated the effects of acute systemic administration of nicotinic acid on sleep in mice. Intraperitoneal and oral gavage administration of nicotinic acid elicited robust increases in non-rapid-eye movement sleep (NREMS) and decreases in body temperature, energy expenditure and food intake. Preventing hypothermia did not affect its sleep-inducing actions suggesting that altered sleep is not secondary to decreased body temperature. Systemic administration of nicotinamide, a conversion product of nicotinic acid, did not affect sleep amounts and body temperature, indicating that it is not nicotinamide that underlies these actions. Systemic administration of monomethyl fumarate, another agonist of the nicotinic acid receptor GPR109A, fully recapitulated the somnogenic and thermoregulatory effects of nicotinic acid suggesting that they are mediated by the GPR109A receptor. The cyclooxygenase inhibitor indomethacin completely abolished the effects of nicotinic acid indicating that prostaglandins play a key role in mediating the sleep and thermoregulatory responses of nicotinic acid.

## Introduction

Metabolic signals play an important role in sleep regulation. Positive energy balance, such as postprandial state, overfeeding and obesity, is accompanied by increased sleep^1–4^, while negative energy states, such as fasting, promote wakefulness^5–8^. Furthermore, hormones that are associated with satiety and/or obesity (*e*.*g*., cholecystokinin, tumor necrosis factor) induce sleep^9,10^, while orexigenic hormones that are associated with fasting and negative energy states (*e*.*g*., ghrelin, orexin), enhance wakefulness^11–13^. Sleep-inducing signals also arise from peripheral metabolic organs, such as the intestines, adipose tissues, liver and skeletal muscle^14–18^.

Sleep is particularly sensitive to shifts in lipid metabolism. The activation of the somatotropic axis, proinflammatory cytokines, bacterial cell wall components, norepinephrine and β3-adrenergic receptor (β3-AR) agonists all stimulate lipolysis, increase circulating levels of free fatty acids (FFAs)^19–22^ and also facilitate sleep^16,23–28^. In nocturnal rodents, sleep is more abundant during the light, lipolytic phase, and wakefulness dominates during the dark, lipogenic phase of the day. Reversal of the lipolytic and lipogenic phases by sequential administration of metabolic hormones^25^ or by restricting feeding to the light phase in rats or mice results in the reversal of the sleep-wake cycle^29,30^. Nicotinic acid, a member of the B3 vitamin group, has been used for over 60 years to improve plasma lipid profile in humans^31,32^. Given the long history of the clinical use of nicotinic acid and its complex actions on lipid metabolism, it is remarkable that its effects on sleep has never been documented.

The acute effects of nicotinic acid intersect with multiple pathways in sleep regulation. In addition to its effects on lipid metabolism, nicotinic acid also markedly stimulates prostaglandin synthesis^33,34^. Prostaglandin D_2_ (PGD_2_) is one of the most potent endogenous sleep-promoting substances^35^. Furthermore, nicotinic acid facilitates the alternative activation of macrophages^36–38^. Alternatively activated macrophages are permissive for maintaining sleep after sleep loss and in cold environment^39^. Nicotinic acid is a potent stimulus for growth hormone secretion^40,41^, and the somatotropic axis has long been implicated in sleep regulation^27^. Finally, butyrate, which binds to the nicotinic acid receptor GPR109A, as well as the short-chain fatty acid receptors FFAR2 and FFAR3, has pronounced sleep-promoting effects^42^.

Considering the reciprocal relationship between sleep and lipid metabolism, the complex actions of nicotinic acid on lipid metabolism and the existence of other multiple regulatory nodes where nicotinic acid may potentially modulate sleep, we investigated the effects of systemic administration of nicotinic acid on sleep. We report that systemic administration of nicotinic acid and another GPR109A receptor agonist, monomethyl fumarate, induces robust sleep and hypothermic responses in mice. Preventing the nicotinic acid-induced hypothermia with β3-AR agonist pretreatment did not attenuate the sleep responses, but the inhibition of prostaglandin synthesis abolished both the sleep and thermoregulatory actions of nicotinic acid.

## Results

### Systemic administration of nicotinic acid elicits robust non-rapid-eye movement sleep (NREMS) increases

**Figure 1:**
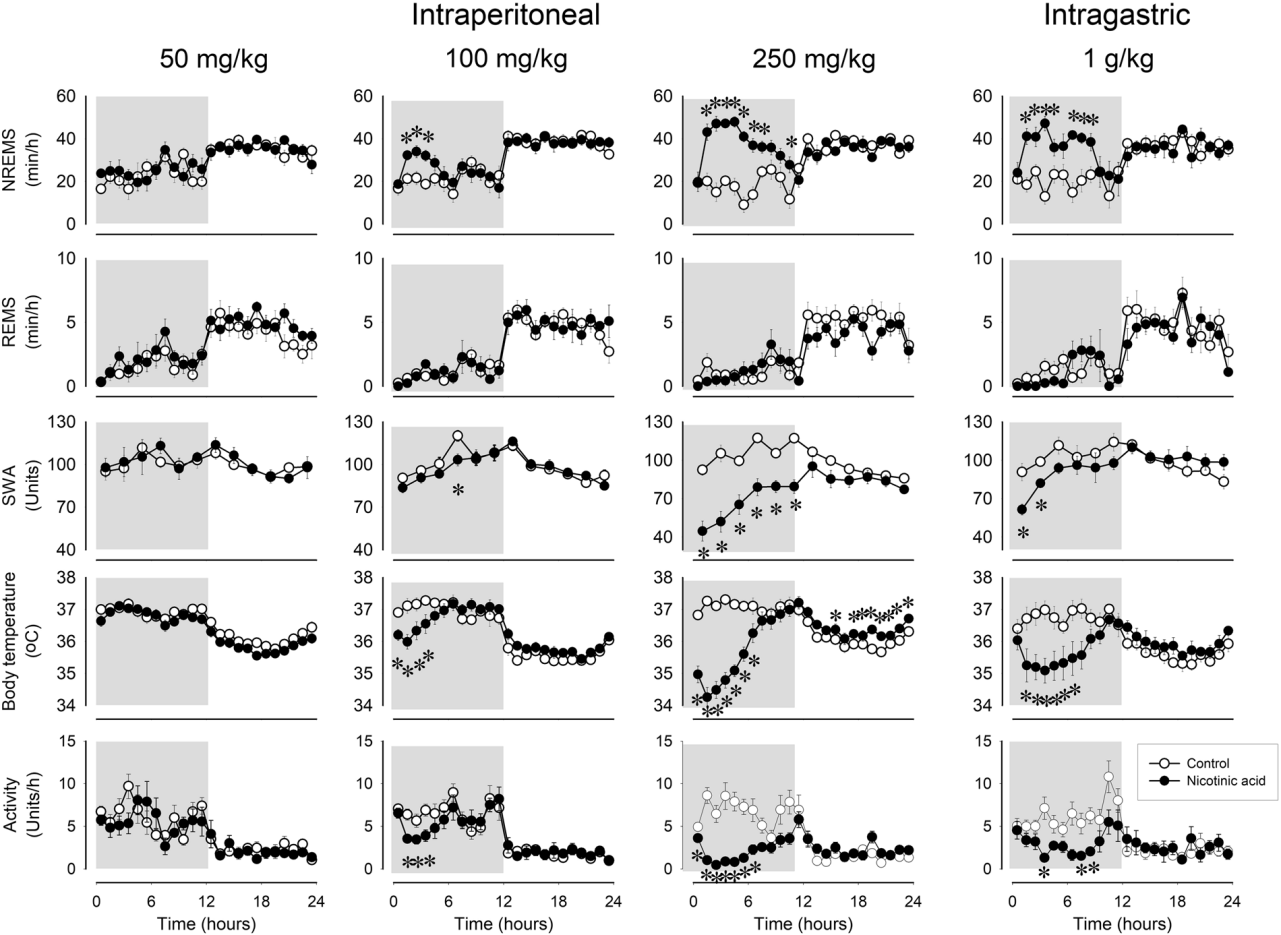
Effects of intraperitoneal (ip) administration of 50, 100 and 250 mg/kg and intragastric delivery of 1 g/kg nicotinic acid on non-rapid-eye movement sleep (NREMS), rapid-eye movement sleep (REMS), electroencephalographic slow wave activity (EEG SWA), body temperature and motor activity in mice. Data are expressed in 1-h time blocks, except EEG SWA values which are shown in 2-h blocks. Nicotinic acid and saline were injected at time “0”. Grey shaded areas represent the dark period. Asterisks: significant difference between control and nicotinic acid treatments, Tukey’s HSD test; error bar: SE.

Intraperitoneal (ip) injection of nicotinic acid brought about marked, dose-dependent increases in NREMS (Fig. 1, Table 1). Intraperitoneal administration of 50 mg/kg nicotinic acid did not affect sleep-wake activity, body temperature or motor activity. The effects of 100 mg/kg nicotinic acid were manifested after a latency of one hour; starting from h 2, NREMS increased 41% above baseline for 4 hours (NREMS amounts in h 2–5, baseline: 82.7 ± 7.3 min, treatment: 116.8 ± 9.6 min, p < 0.01). Administration of 250 mg/kg nicotinic acid elicited robust and long-lasting increases in NREMS; NREMS was elevated 120% above baseline in the 2–11 h time block (baseline: 179.0 ± 7.1 min, treatment: 394.6 ± 20.4 min, p < 0.001). The effects of oral administration of 1 g/kg nicotinic acid were similar to those of the highest ip dose. NREMS increased by 86% in the 2–11 h time block, then returned to baseline in the latter half of the recording period (baseline: 197.9 ± 8.1 min, treatment: 368.9 ± 25.0 min, p < 0.001). NREMS increases were accompanied by decreased electroencephalographic (EEG) slow-wave activity (SWA) and suppressed motor activity (Fig. 1, Table 1).

**Table 1:** The effects of nicotinic acid on non-rapid-eye movement sleep (NREMS), rapid-eye movement sleep (REMS), body temperature, motor activity and electroencephalographic slow-wave activity (EEG SWA): statistical results.

	NREMS			REMS			Temperature			Activity			SWA		
	*df*	*F*	*p*	*df*	*F*	*p*	*df*	*F*	*p*	*df*	*F*	*p*	*df*	*F*	*p*
**50** **mg/kg**, **ip**															
*Treatment*	1,6	0.6	n.s.	1,6	12.6	p < 0.05	1,6	13.9	p < 0.01	1,6	1.7	n.s.	1,6	0.2	n.s.
*Time*	23,138	6.3	p < 0.001	23,138	7.7	p < 0.001	23,138	28.5	p < 0.001	23,138	7.4	p < 0.001	11,66	1.5	n.s.
*Treatment × Time*	23,138	1.2	n.s.	23,138	0.9	n.s.	23,138	1.0	n.s.	23,138	1.3	n.s.	11,66	0.8	n.s.
***100*** ***mg/kg***, ***ip***															
*Treatment*	1,7	13.6	p < 0.01	1,7	0.0	n.s.	1,7	0.0	n.s.	1,7	10.0	p < 0.05	1,7	4.2	n.s.
*Time*	23,161	14.7	p < 0.001	23,161	17.8	p < 0.001	23,161	27.9	p < 0.001	23,161	16.9	p < 0.001	11,77	9.9	p < 0.001
*Treatment × Time*	23,161	1.5	n.s.	23,161	0.9	n.s.	23,161	4.5	p < 0.001	23,161	1.2	n.s.	11,77	2.0	p < 0.05
***250*** ***mg/kg***, ***ip***															
*Treatment*	1,6	95.3	p < 0.001	1,6	8.2	p < 0.05	1,6	26.2	p < 0.01	1,6	87.3	p < 0.001	1,6	18.0	p < 0.01
*Time*	23,138	8.9	p < 0.001	23,138	15.2	p < 0.001	23,138	15.7	p < 0.001	23,138	10.1	p < 0.001	11,66	14.3	p < 0.001
*Treatment × Time*	23,138	9.3	p < 0.001	23,138	1.4	n.s.	23,138	35.2	p < 0.001	23,138	8.0	p < 0.001	11,66	16.5	p < 0.001
***1***,***000*** ***mg/kg***, ***oral gavage***															
*Treatment*	1,4	23.3	p < 0.01	1,4	2.1	n.s.	1,4	4.2	n.s.	1,5	19.2	p < 0.01	1,4	1.2	n.s.
*Time*	23,92	3.4	p < 0.001	23,92	11.0	p < 0.001	23,92	3.7	p < 0.001	23,115	5.2	p < 0.001	11,44	4.3	p < 0.001
*Treatment × Time*	23,92	5.2	p < 0.001	23,92	1.5	n.s.	23,92	5.9	p < 0.001	23,115	3.2	p < 0.001	11,44	5.1	p < 0.001

Nicotinic acid had only minor effects on rapid-eye movement sleep (REMS) without any apparent dose-dependency. Oral gavage administration of 1 g/kg or ip injection of 100 mg/kg nicotinic acid did not affect REMS, while 50 and 250 mg/kg had a slight, but statistically significant effect (Table 1, Fig. 1). After 50 mg/kg nicotinic acid, REMS was elevated in h 21–23, while after 250 mg/kg, it was slightly below baseline in h 12 and 20.

On the recovery days following the ip administration of 250 mg/kg or the gavage administration of 1 g/kg nicotinic acid, sleep-wake activity returned to baseline with no significant rebound responses (for ip 250 mg/kg nicotinic acid, wakefulness baseline: 482.2 ± 9.1 and 208.6 ± 7.8 min/12 h during the dark and light periods, respectively, recovery day: 460.7 ± 17.7 and 201.1 ± 4.5 min/12 h during the dark and light periods, respectively; NREMS baseline: 224.5 ± 8.7 and 449.1 ± 7.8 min/12 h, recovery day: 240.9 ± 15.1 and 452.9 ± 4.6 min/12 h; REMS baseline: 13.3 ± 1.7 and 62.4 ± 5.5 min /12 h, recovery day: 18.4 ± 3.1 and 65.9 ± 1.8 min/12 h; for gavage 1 g/kg nicotinic acid, wakefulness baseline: 471.0 ± 15.6 and 217.8 ± 14.7 min/12 h during the dark and light periods, respectively, recovery day: 460.5 ± 20.5 and 214.4 ± 21.6 min/12 h during the dark and light periods, respectively; NREMS baseline: 235. 7 ± 11.3 and 443.2 ± 15.8 min/12 h, recovery day: 241.9 ± 14.8 and 447.6 ± 18.2 min/12 h; REMS baseline: 13.3 ± 4.9 and 59.1 ± 4.7 min /12 h, recovery day: 11.7 ± 3.5 and 57.9 ± 7.0 min/12 h).

The sleep responses were paralleled by significant suppressions in body temperature. Body temperature decreased by ~1 °C for 4 h after the 100 mg/kg dose. In response to 250 mg/kg nicotinic acid, the maximum of the hypothermic response reached 3 °C in the second hour after the treatment (Fig. 1, Table 1). The average body temperature in the 2–11 h time period, when NREMS increases were significant, was 1.34 ± 0.2 °C below baseline (p < 0.001). During the second half of the recording period temperatures increased slightly, but significantly, above baseline. The effects of oral administration of 1 g/kg nicotinic acid on body temperature were similar, but somewhat attenuated as compared to the effects of the highest ip dose.

### Indomethacin blocks the effects of nicotinic acid on sleep and body temperature

**Figure 2:**
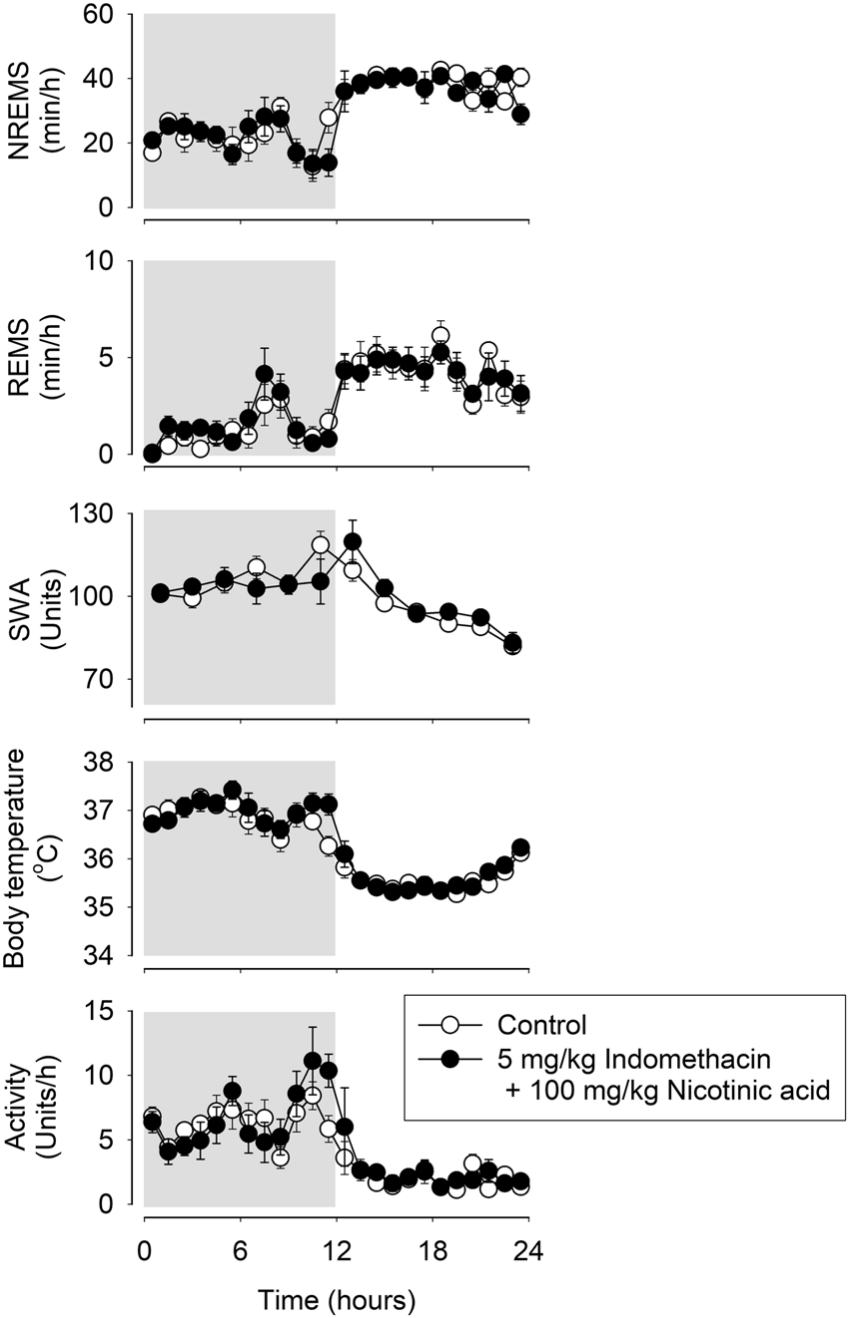
Effects of 5 mg/kg indomethacin pretreatment on the NREMS, REMS, EEG SWA, motor activity and body temperature-modulating actions of 100 mg/kg nicotinic acid in mice. See legend to Fig. 1 for details.

To examine the role of endogenous prostaglandins in the sleep and thermoregulatory effects of nicotinic acid, mice were pretreated with 5 mg/kg indomethacin, a cyclooxygenase inhibitor, before the administration of 100 mg/kg nicotinic acid. Indomethacin pretreatment completely abolished the effects of nicotinic acid on sleep, EEG SWA, body temperature and motor activity (Fig. 2, Table 2). Indomethacin, when administered by itself, did not have any significant effect on sleep and body temperature (data not shown).

**Table 2:** Indomethacin pretreatment followed by administration of nicotinic acid. NREMS, REMS, body temperature, motor activity and EEG SWA: statistical results.

	NREMS			REMS			Temperature			Activity			SWA		
	*df*	*F*	*p*	*df*	*F*	*p*	*df*	*F*	*p*	*df*	*F*	*p*	*df*	*F*	*p*
*Treatment*	1,6	0.4	n.s.	1,6	0.9	n.s.	1,6	1.2	n.s.	1,6	0.7	n.s.	1,6	0.2	n.s.
*Time*	23,138	14.4	p < 0.001	23,138	12.3	p < 0.001	23,138	44.7	p < 0.001	23,138	10.8	p < 0.001	11,66	10.7	p < 0.001
*Treatment × Time*	23,138	1.2	n.s.	23,138	0.5	n.s.	23,138	1.3	n.s.	23,138	1.0	n.s.	11,66	1.3	n.s.

### Decreased body temperature is not the cause of nicotinic acid-induced sleep

**Figure 3:**
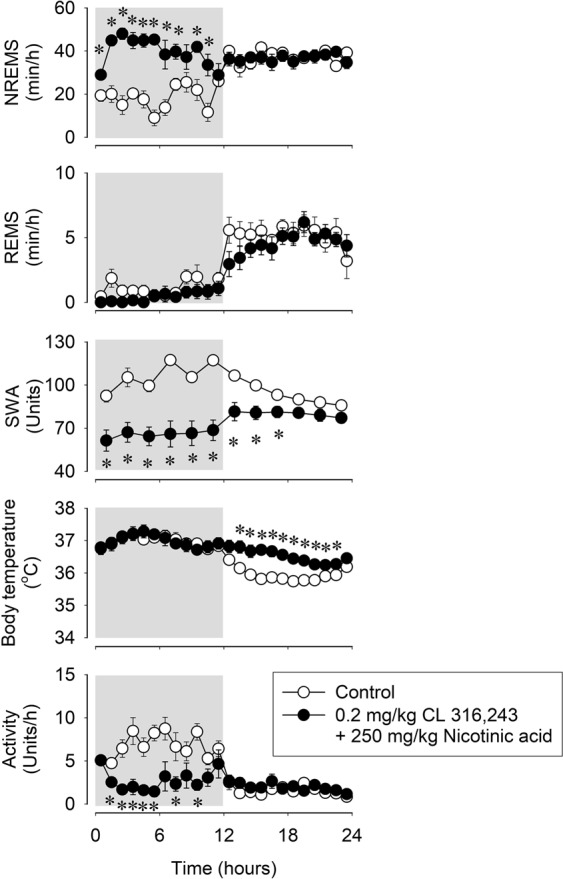
Effects of the β3-adrenergic receptor agonist, CL-316,243, pretreatment on the NREMS, REMS, EEG SWA, motor activity and body temperature-modulating actions of 250 mg/kg nicotinic acid in mice. See legend to Fig. 1 for details.

To investigate if decreased body temperature, *per se*, could account for increased sleep in response to nicotinic acid, mice were pretreated with CL-316,243, a β3-AR agonist, to stimulate thermogenesis thereby preventing hypothermia. CL-316,243 pretreatment completely abolished the hypothermic response to 250 mg/kg nicotinic acid but did not attenuate the late increases in body temperature in the second half of the recording period (Table 3, Fig. 3). The NREMS-promoting effects of nicotinic acid were not attenuated by the β3-AR agonist pretreatment, and sleep was increased by 134% in the 2–11 h time block (NREMS amounts in the 2–11 h time block; baseline: 179.0 ± 7.1 min, CL-316,243 + nicotinic acid treatment: 394.6 ± 20.4 min, p < 0.001). Likewise, the motor activity and EEG SWA suppressions after nicotinic acid administration were not affected by the CL-316,243 pretreatment.

**Table 3:** CL-316,243 pretreatment followed by administration of nicotinic acid. NREMS, REMS, body temperature, motor activity EEG SWA: statistical results.

	NREMS			REMS			Temperature			Activity			SWA		
	*df*	*F*	*p*	*df*	*F*	*p*	*df*	*F*	*p*	*df*	*F*	*p*	*df*	*F*	*p*
*Treatment*	1,6	65.3	p < 0.001	1,6	13.4	p < 0.05	1,6	17.4	p < 0.01	1,6	22.8	p < 0.01	1,6	28.4	p < 0.01
*Time*	23,138	4.2	p < 0.001	23,138	21.5	p < 0.001	23,138	25.7	p < 0.001	23,138	9.2	p < 0.001	11,66	3.2	p < 0.01
*Treatment × Time*	23,138	9.2	p < 0.001	23,138	0.8	n.s.	23,138	3.1	p < 0.001	23,138	5.4	p < 0.001	11,66	11.9	p < 0.001

### Systemic administration of nicotinamide does not affect sleep amounts and body temperature

**Figure 4:**
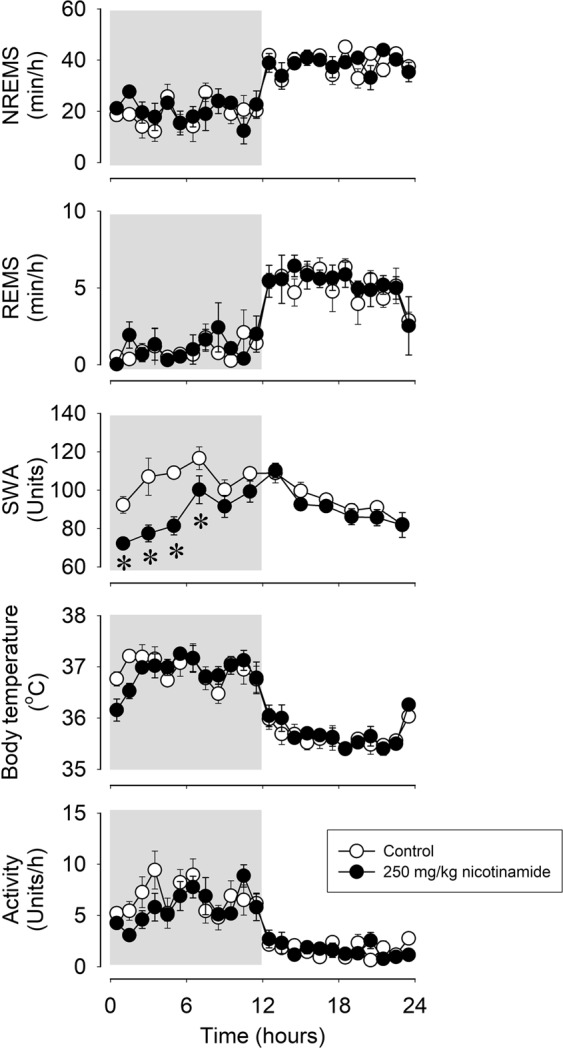
Effects of ip administration of 250 mg/kg nicotinamide on NREMS, REMS, EEG SWA, motor activity and body temperature in mice. See legend to Fig. 1 for details.

To test the possibility that its conversion into nicotinamide underlies the sleep-promoting effects of nicotinic acid, we investigated the effects of nicotinamide on sleep and body temperature. Systemic injection of 100 mg/kg (data not shown) or 250 mg/kg nicotinamide did not have any effect on sleep time and body temperature (Fig. 4, Table 4). The higher dose slightly decreased motor activity and suppressed EEG SWA.

**Table 4:** The effects of 250 mg/kg nicotinamide on NREMS, REMS, body temperature, motor activity and EEG SWA: statistical results.

	NREMS			REMS			Temperature			Activity			SWA		
	*df*	*F*	*p*	*df*	*F*	*p*	*df*	*F*	*p*	*df*	*F*	*p*	*df*	*F*	*p*
*Treatment*	1,4	0.1	n.s.	1,4	0.8	n.s.	1,4	0.0	n.s.	1,4	12.1	p < 0.05	1,4	419.3	p < 0.001
*Time*	23,92	15.6	p < 0.001	23,92	18.7	p < 0.001	23,92	32.9	p < 0.001	23,92	12.2	p < 0.001	11,44	4.3	p < 0.001
*Treatment × Time*	23,92	1.2	n.s.	23,92	0.6	n.s.	23,92	1.1	n.s.	23,92	1.2	n.s.	11,44	4.8	p < 0.001

### Monomethylfumarate induces NREMS

**Figure 5:**
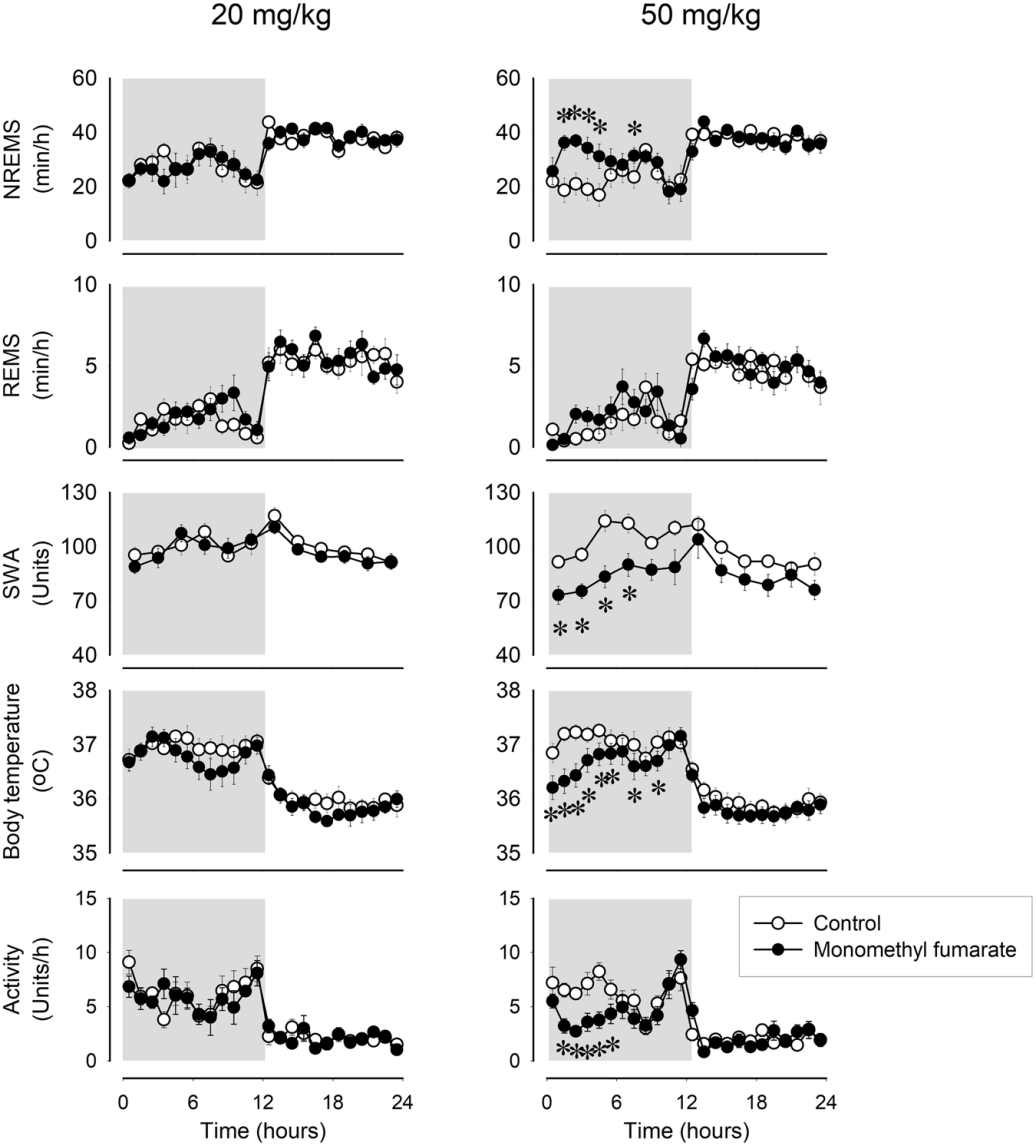
Effects of ip administration of 20 and 50 mg/kg monomethyl fumarate on NREMS, REMS, EEG SWA, motor activity and body temperature in mice. See legend to Fig. 1 for details.

To further investigate the involvement of the nicotinic acid receptor, GPR109A, in the sleep and thermoregulatory responses, we studied the effects of monomethyl fumarate (MMF), another GPR109A agonist. Administration of MMF resulted in sleep and body temperature responses similar to those of nicotinic acid. The lowest effective dose to induce sleep was 50 mg/kg, which elicited 84% increase in NREMS during the 4-h time block starting from h 2 (NREMS amounts in h 2–5, baseline: 75.4 ± 11.8 min, treatment: 138.4 ± 6.8 min, p < 0.001; Fig. 5, Table 5). The sleep effects were accompanied by a 0.6–0.9 °C drop in body temperature, suppressed motor activity and EEG SWA. A lower dose of MMF, 20 mg/kg, did not have significant effects on sleep and body temperature.

**Table 5:** Intraperitoneal administration of monomethyl fumarate. NREMS, REMS, body temperature, motor activity and EEG SWA: statistical results.

	NREMS			REMS			Temperature			Activity			SWA		
	*df*	*F*	*p*	*df*	*F*	*p*	*df*	*F*	*p*	*df*	*F*	*p*	*df*	*F*	*p*
***20*** ***mg/kg***															
*Treatment*	1,7	0.4	n.s.	1,7	3.1	n.s.	1,7	21.3	p < 0.01	1,7	0.6	n.s.	1,7	0.9	n.s.
*Time*	23,161	7.0	p < 0.001	23,161	17.5	p < 0.001	23,161	23.3	p < 0.001	23,161	8.1	p < 0.001	11,77	3.8	p < 0.001
*Treatment × Time*	23,161	1.0	n.s.	23,161	1.0	n.s.	23,161	1.1	n.s.	23,161	0.8	n.s.	11,77	1.1	n.s.
***50*** ***mg/kg***															
*Treatment*	1,7	17.3	p < 0.01	1,7	9.8	p < 0.05	1,8	10.7	p < 0.05	1,8	16.5	p < 0.01	1,7	8.8	p < 0.05
*Time*	23,161	8.3	p < 0.001	23,161	13.0	p < 0.001	23,184	23.2	p < 0.001	23,184	12.9	p < 0.001	11,77	6.6	p < 0.001
*Treatment × Time*	23,161	3.1	p < 0.001	23,161	1.6	n.s.	23,184	3.3	p < 0.001	23,184	2.7	p < 0.001	11,77	2.0	p < 0.05

### Nicotinic acid suppresses VO_2_, respiratory exchange ratio (RER) and feeding

**Figure 6:**
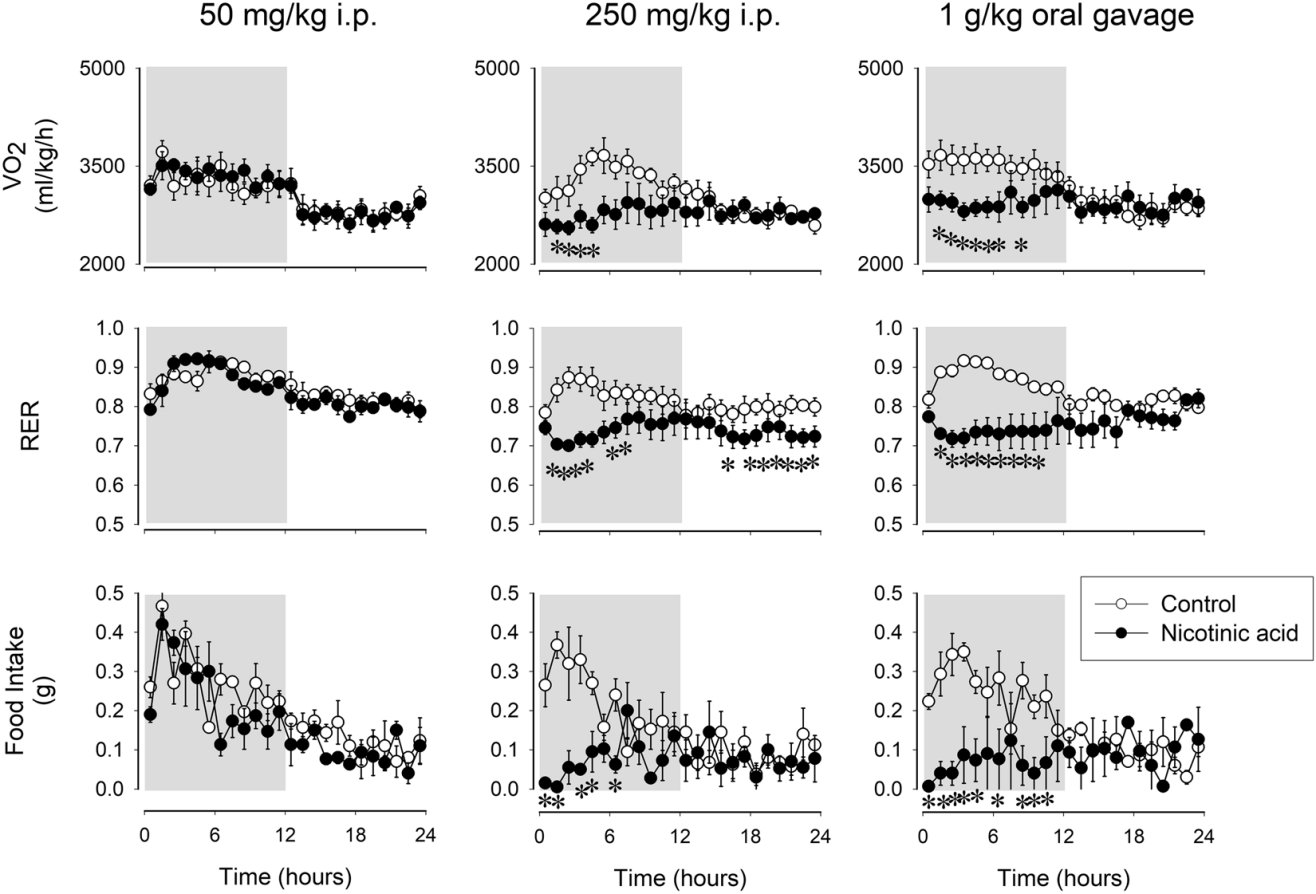
Effects of ip administration of 50 and 250 mg/kg and intragastric delivery of 1 g/kg nicotinic acid on oxygen consumption (VO_2_), respiratory exchange ratio (RER) and food intake in mice. Data are expressed in 1-h time blocks. See legend to Fig. 1 for details.

To gain further insight in the action of somnogenic doses of nicotinic acid on metabolism, we investigated the effects of ip and oral administration of nicotinic acid on O_2_ consumption (VO_2_), RER and food intake. Intraperitoneal injection of 50 mg/kg nicotinic acid did not have any effect on these measures, while 250 mg/kg nicotinic acid induced marked and long-lasting suppression of VO_2_, RER and feeding. Similar robust effects were observed after gavage administration of 1 g/kg nicotinic acid (Fig. 6, Table 6).

**Table 6:** Effects of nicotinic acid on VO_2_, RER and food intake: statistical results.

	VO_2_			RER			Food intake		
	*df*	*F*	*p*	*df*	*F*	*p*	*df*	*F*	*p*
*Treatment*	1,3	3.2	n.s.	1,3	3.4	n.s.	1,3	6.9	n.s.
*Time*	23,69	17.7	p < 0.001	23,69	8.9	p < 0.001	23,69	9.1	p < 0.001
*Treatment × Time*	23,69	1.1	n.s.	23,69	1.0	n.s.	23,69	1.4	n.s.
***250*** ***mg/kg***, ***ip***									
*Treatment*	1,3	5.5	n.s.	1,3	39.7	p < 0.01	1,3	10.4	p < 0.05
*Time*	23,69	4.7	p < 0.001	23,69	1.8	p < 0.05	23,69	12.5	p < 0.01
*Treatment × Time*	23,69	2.8	p < 0.001	23,69	1.5	n.s.	23,69	3.8	p < 0.001
***1*** ***g/kg***, ***oral gavage***									
*Treatment*	1,3	6.4	n.s.	1,3	9.1	0.057	1,3	29.9	p < 0.05
*Time*	23,69	7.3	p < 0.001	23,69	1.6	n.s.	23,69	2.9	p < 0.001
*Treatment × Time*	23,69	4.4	p < 0.001	23,69	4.5	p < 0.001	23,69	7.8	p < 0.001

## Discussion

Nicotinic acid was introduced into clinical therapy more than 60 years ago to improve plasma lipid profile, but its effects on sleep and body temperature have never been documented. Our main finding is that systemic injection or oral gavage administration of nicotinic acid induces robust increases in NREMS in mice. The effects were dose-dependent, ip administration of 100 and 250 mg/kg doses increased NREMS 41–120% above baseline for 4–11 h. Oral administration of 1 g/kg nicotinic acid had similar sleep-promoting potency as the 250 mg/kg injected ip. The effects were specific to NREMS, as REMS responses were minor and did not show dose-dependency.

Nicotinic acid is part of a group known as B3 complex, which also includes nicotinamide and nicotinamide riboside. After ip administration, 25% of the injected nicotinic acid is converted to nicotinamide in the liver in mice^43^. To determine if the acute sleep-promoting actions of nicotinic acid could be due to its conversion to nicotinamide, we also investigated the effects of bolus injection of nicotinamide. Intraperitoneal injection of 100 and 250 mg/kg nicotinamide did not have any significant effect on sleep amounts or body temperature, thus, it is unlikely that the sleep-promoting and thermoregulatory actions of nicotinic acid are mediated by its conversion to nicotinamide. Further, portions of circulating nicotinic acid and nicotinamide are converted to nicotinamide adenine dinucleotide (NAD) in the liver^44^. It has been reported that metabolic signals from the hepatoportal region modulate sleep^17,42^. Since the conversion of nicotinamide to NAD is significantly more efficient than that of nicotinic acid^45^, but nicotinamide lacks sleep-inducing activity, it is unlikely that nicotinic acid-induced increases in liver NAD content are responsible for the sleep-promoting effects.

Nicotinic acid induced significant decreases in body temperature. This can be explained, in part, by increased heat loss through cutaneous vasodilation. Nicotinic acid-induced vasodilation has been described in guinea pigs^46^, rats^47^ and mice^34^ and it is the cause of flushing, the well-known side effect of nicotinic acid megadoses in humans^48^. Vasodilation is a prompt and transient response to nicotinic acid^48–50^. In mice, vasodilation induced by subcutaneous injection of 100–300 mg/kg nicotinic acid reaches a peak within 2–3 min, and by 10 min the effect is reduced by 50%^34^. The sleep-inducing and the hypothermic actions of nicotinic acid, however, lasted for ~7–11 hours, thus it is unlikely that they are the direct consequences of the actions of nicotinic acid on cutaneous blood flow.

For thermoregulation, mice rely more on metabolic thermogenesis rather than vasomotor mechanisms^51^, and it is likely that the hypothermia is due to suppressed metabolic heat production. In fact, metabolic heat production (VO_2_) was suppressed in response to nicotinic acid in our study. Consistent with our finding, 200 mg/kg nicotinic acid attenuates cold-induced increases in thermogenesis in mice^52^ and norepinephrine-induced^53^ or spontaneous^54^ thermogenesis in rats. A major factor contributing to decreased thermogenesis is suppressed feeding in mice. Food intake was reduced by ~75% after 250 mg/kg ip and 1 g/kg oral doses of nicotinic acid in the first 8 hours, when VO_2_ and sleep responses were the most pronounced. Similar reductions in feeding were also reported after repeated nicotinic acid treatment in mice^55^. Since naturally occurring NREMS is associated by decreased energy expenditure and body temperature^56^, it is also possible that the hypothermic response is simply the thermic manifestation of enhanced NREMS after nicotinic acid treatment. Similar robust ~4 °C hypothermia was observed during enhanced sleep in response to the chemogenetic stimulation of the ventrolateral preoptic sleep center^57^.

There is a complex relationship between sleep and feeding. For example, sleep loss increases feeding^58,59^ and increased food intake induces postprandial sleep^2,3^. Since short-term food deprivation elicits robust increases in wakefulness and motor activity in mice^5–7^, it is unlikely that increased sleep and suppressed motor activity after nicotinic acid treatment are regulated energy-saving mechanisms in response to suppressed caloric intake. It is possible that the primary effect of nicotinic acid is increased sleep, and since sleep and feeding are mutually exclusive behaviors, decreased feeding may be the consequence of the general behavioral inactivity. Alternatively, the effects of nicotinic acid on sleep and feeding may be independent and mediated by different mechanisms.

It has been proposed that changes in body temperature may trigger somnogenic brain areas to initiate sleep^60^. To ascertain whether hypothermia *per se* could be a factor in the sleep-inducing effects of nicotinic acid, we also tested the effects in mice after the stimulation of thermogenesis by CL-316,243. CL-316,243 pretreatment completely abolished the hypothermic response, but the NREMS-promoting effects of nicotinic acid were not attenuated indicating that decreased body temperature, by itself, cannot account for the somnogenic effects of nicotinic acid.

Most, but not all, of the effects of nicotinic acid are mediated by GPR109A, the high-affinity nicotinic acid receptor^61–63^. To explore the role of the receptor in the sleep and thermoregulatory responses to nicotinic acid, we also used another receptor agonist, MMF^64^. MMF treatment fully recapitulated the effects of nicotinic acid on sleep, EEG SWA, body temperature and motor activity. Further, butyrate, another ligand for the GPR109A receptors as well as for the FFAR2, FFAR3 receptors, has similar marked NREMS-promoting and hypothermic effects as nicotinic acid^42^. These findings are consistent with the notion that the effects on sleep and temperature are mediated by the GPR109A receptor.

Nicotinic acid and MMF are potent stimuli for prostaglandin production via the activation of GPR109A receptors^65–67^. Ingestion of nicotinic acid results in 400 to 800-fold increases in PGD_2_ plasma levels in humans^33^, and systemic injection of 100–300 mg/kg leads to 1.5–4-fold increases in mice^34^. Since PGD_2_ has been identified as one of the most potent endogenous sleep-promoting molecules^35^, we sought to assess the role of prostaglandins in nicotinic acid-induced sleep by using the cyclooxygenase inhibitor indomethacin. Indomethacin pretreatment completely abolished the sleep-promoting as well as thermoregulatory actions of nicotinic acid indicating the involvement of endogenous prostaglandins both in the somnogenic and hypothermic effects. The only known significant sources of nicotinic acid-induced prostaglandin production are Langerhans cells and keratinocytes in the skin^66,67^. The ventral surface of the rostral basal forebrain has been identified as a PGD_2_-sensitive sleep-promoting site, and it is possible that peripherally produced prostaglandins reach this structure. Alternatively, prostaglandins may act on a peripheral target to induce sleep. For example, vagus afferents express EP3 and EP4 prostaglandin receptors^68,69^ and prostaglandins activate vagal sensory nerves^70–72^. It is known that vagal afferents carry sleep-inducing signals^73–76^.

Nicotinic acid, unlike nicotinamide, does not readily pass between blood and the cerebrospinal fluid^77–79^ and the relatively small amounts that enter the brain are promptly converted to nicotinamide^77^. Therefore, it is less likely that centrally-produced prostaglandins underly the sleep-inducing actions of nicotinic acid, although this possibility cannot be ruled out with certainty.

Nicotinic acid has complex actions on lipid metabolism. It is considered an antilipolytic agent because it suppresses FFA release from adipocytes *in vitro* and causes transient decreases in circulating FFA levels *in vivo*^31^. However, decreased FFA levels are followed by robust rebound increases 2–4 h after a single bolus treatment in humans^80–82^, dogs^83^ and rats^83,84^. Since the antilipolytic actions of nicotinic acid are independent of prostaglandins^85^, but the sleep-promoting actions require intact prostaglandin synthesis, it is unlikely that the antilipolytic actions of nicotinic acid directly contribute to its sleep effects. Independent of its lipolytic actions, nicotinic acid also promotes complex changes in plasma lipid profile, an action that is thought to underlie its antiatherogenic properties^86^. It cannot be ruled out that these changes may also contribute to metabolic signaling for sleep.

The extent to which our findings in mice are also applicable to humans is not known. It has been argued that the dose range used in our study corresponds well to the doses used in humans, if the 10-times faster metabolism of mice as compared to humans is considered^87^. The use of nicotinic acid supplements is widespread in the United States, and they are often advertised as sleep aids. In human trials, the most common reason for withdrawal is flushing followed by fatigue/sleepiness^88–91^. Our findings together with the above observational evidence point to the need of characterizing the effects of nicotinic acid on sleep in humans.

## Methods

### Animals

All procedures involving the use of animals followed the recommendations of the Guide for the Care and Use of Laboratory Animals of the National Institutes of Health. All animal husbandry and experimental procedures were carried out in accordance with the Association for Assessment and Accreditation of Laboratory Animal Care (AAALAC) and approved by the Institutional Animal Care and Use Committee (IACUC) of the Washington State University (protocol number 6031). Breeding pairs of C57BL/6J mice were purchased from The Jackson Laboratories, Inc. and were bred in-house at Washington State University. During the experiments, the animals were housed individually in temperature-controlled (29 ± 1 °C), sound-attenuated environmental chambers on a 12:12-hour light-dark cycle (lights on at 3 AM). Food (Harlan Teklad, Product no. 2016) and water were available unrestricted throughout all experiments.

### Surgery

Male mice were surgically instrumented using ketamine-xylazine anesthesia (87 and 13 mg/kg, respectively). For sleep-wake activity recordings three cortical EEG electrodes, placed over the frontal and parietal cortices, and two nuchal electromyographic (EMG) electrodes were implanted. Leads from the EEG and EMG electrodes were anchored to the skull with dental cement. Telemetry transmitters were implanted ip for body temperature and motor activity recordings (Starr Life Sciences Corp., model G2 Emitter). The weight of a G2 Emitter is 1.1 g. The animals were allowed to recover from surgery for at least 10 days before any experimental manipulation started and handled daily to adapt them to the experimental procedures. The body weight of the mice at the time of the experiment was 28.9 ± 0.5 g.

### Sleep-wake activity recordings and analyses

The animals were connected to the recording system through a lightweight, flexible tether plugged into a commutator, which was further routed to Grass Model 15 Neurodata amplifier system (Grass Instrument Division of Astro-Med, Inc., West Warwick, RI). The amplified EEG and EMG signals were digitized at 256 Hz and recorded by computer. The high-pass and low-pass filters for EEG signals were 0.5 and 30.0 Hz, respectively. The EMG signals were filtered with low and high cut-off frequencies at 100 and 10,000 Hz, respectively. The outputs from the 12A5 amplifiers were fed into an analog-to-digital converter and collected by computer using SleepWave software (Biosoft Studio, Hersey, PA). Sleep-wake states were scored visually off-line in 10-s segments. The vigilance states were defined as NREMS, REMS and wakefulness according to standard criteria as described previously^92^. Artifact-free EEG epochs were subjected to off-line spectral analysis by fast Fourier transformation. EEG power data in the range of 0.5 to 4.0 Hz during NREMS were used to compute EEG SWA. EEG SWA data were normalized for each animal by using the average EEG SWA across 24 h on the baseline day as 100.

### Telemetry recordings

Core body temperature and locomotor activity were recorded by MiniMitter telemetry system (Starr Life Sciences Corp., model G2 Emitter and ER-4000 receiver) using VitalView software. Temperature and activity values were collected every 1 and 10 min, respectively, throughout the experiment and were averaged over 1-h time blocks.

### Experimental procedures

#### Experiment 1: The effects of systemic administration of nicotinic acid on sleep-wake activity, body temperature and metabolic parameters in mice

Three groups of male mice were habituated to the injection procedure by administering 0.3 ml isotonic saline daily for 7 days 5–10 min before dark onset. On the baseline days, the animals were injected ip with 10 ml/kg isotonic NaCl. On the test day, the animals received 50, 100 or 250 mg/kg nicotinic acid (Millipore Sigma) in a volume of 10 ml/kg (n = 6, n = 8, n = 7, respectively). The treatments were performed 5–10 min before dark onset. Sleep and telemetry recordings started at the onset of the dark phase and continued for 24 h.

Another group of mice (n = 5) was habituated to the gavage procedure by administering 0.3 ml water daily for 7 days 5–15 min before dark onset. After the habituation period, a baseline day was recorded after the oral gavage of 0.3 ml vehicle. The following day, 1 g/kg nicotinic acid was administered in the volume of 0.3 ml. The treatments were performed 5–10 min before dark onset. Sleep and telemetry recordings started at the onset of the dark phase and continued for 24 h.

#### Experiment 2: The effects of indomethacin pretreatment on the sleep-wake activity and body temperature-modulating effects of nicotinic acid in mice

A group of mice (n = 7) was habituated to the injection procedure as described above. On the baseline day, the animals were injected ip with 10 ml/kg isotonic NaCl. On the test day, the animals were pretreated with 5 mg/kg indomethacin subcutaneously 30 min prior the beginning of the dark phase, followed by 100 mg/kg nicotinic acid ip injection 5 minutes before dark onset. Sleep and telemetry recordings started at onset of the dark phase and continued for 24 h.

#### Experiment 3: The effects of β3-adrenergic receptor agonist, CL-316,243, pretreatment on the sleep-wake activity and body temperature-modulating effects of nicotinic acid in mice

A group of mice (n = 7) was habituated to the injection procedure as described above. On the baseline days, the animals were injected with 10 ml/kg isotonic NaCl ip. On the test day, the animals were pretreated with subcutaneous 0.2 mg/kg CL-316,243 (Millipore Sigma) 30 min prior the beginning of the dark phase, followed by ip 250 mg/kg nicotinic acid injection 5 minutes before dark onset. Sleep and telemetry recordings started at onset of the dark phase and continued for 24 h.

#### Experiment 4: The effects of ip administration of nicotinamide in mice

Two doses of nicotinamide were tested in the same group of mice (n = 6). After the habituation period, the animals received 10 ml/kg isotonic NaCl ip to obtain baseline values. On the test day, the mice were injected with 100 mg/kg nicotinamide ip. Two days later, a new baseline day was recorded followed by the test day of 250 mg/kg nicotinamide. The treatments were performed 5–10 min before dark onset. Sleep and telemetry recordings started at onset of the dark phase and continued for 24 h.

#### Experiment 5: The effects of ip administration of monomethyl fumarate in mice

Ten male mice were habituated to the injection procedure by daily administration of isotonic saline 5–10 min before dark onset. On the baseline days, the animals were injected ip with 10 ml/kg isotonic NaCl. On the test day, the animals received 20 or 50 mg/kg monomethyl fumarate in a volume of 10 ml/kg (n = 8, for both; five mice were injected with both doses of MMF). The treatments were at least two days apart and performed 5–10 min before dark onset. Sleep and telemetry recordings started at onset of the dark phase and continued for 24 h.

#### Experiment 6: The effects of nicotinic acid on energy expenditure and food intake in mice

Oxygen consumption (VO_2_, ml/kg/h) and RER were measured via indirect calorimetry; food intake was measured simultaneously by an automated system (Oxymax System, Columbus Instruments, Columbus, OH). Animals were habituated to the calorimetry cages for at least three days before the recordings. On the baseline day, vehicle was administered, on the test days 50 mg/kg (n = 4) or 250 mg/kg (n = 4) nicotinic acid was injected ip, or 1 g/kg nicotinic acid was administered via oral gavage (n = 4). All treatments were performed 5–10 min before the onset of the dark period.

### Statistics

Time spent in wakefulness, NREMS and REMS, as well as body temperature and motor activity, VO_2_ and RER were calculated in 1-h blocks, and EEG SWA was calculated in 2-h blocks. Two-way repeated measures ANOVA was performed across 24 h between test days and the corresponding baselines (factors: treatment and time, both repeated). When appropriate, Tukey’s HSD test was applied post hoc. An α-level of P < 0.05 was considered to be significant.

## Data Availability

The datasets generated during and/or analyzed during the current study are available from the corresponding author on reasonable request.
